# Retrograde jejunoduodenogastric intussusception associated with fully functioning nonballoon nasojejunal feeding catheter: A case report

**DOI:** 10.1097/MD.0000000000037772

**Published:** 2024-04-12

**Authors:** Seung Soo Lee

**Affiliations:** a Department of Surgery, School of Medicine, Kyungpook National University, Daegu, Republic of Korea; b Department of Surgery, Kyungpook National University Hospital, Daegu, Republic of Korea.

**Keywords:** Case report, catheters, enteral nutrition, gastroesophageal reflux, intussusception

## Abstract

**Rationale:**

Retrograde jejunoduodenogastric intussusception refers to invagination of distal small intestine into the stomach. It is extremely rare. It is often associated with displaced feeding catheter in which its balloon tip migrates past the gastric pylorus. The intussusception is triggered by retraction of migrated catheter. It is often accompanied by feeding intolerance or catheter malfunction. This report describes a distinctive case of retrograde jejunoduodenogastric intussusception associated with a fully functioning nonballoon nasojejunal tube.

**Patient concern:**

A 19-year-old female was presented with repeated vomiting and abdominal distension for 5 days.

**Diagnosis:**

An abdominal computerized tomography revealed retrograde jejunoduodenogastric intussusception causing air/fluid-filled gastric distension. Immediate endoscopic examination revealed a loop of small intestine, protruding through the pylorus. Progressed ischemia of the migrated small bowel loop was confirmed.

**Interventions:**

At laparotomy, a jejunal loop migrating into the duodenum and stomach at the level of the ligament of Treitz was noticed. After manual reduction of migrated bowel, 2 segmental resections of necrotic segment were performed. A feeding jejunostomy was constructed in the proximal jejunum.

**Outcomes:**

Enteral feeding through the surgically constructed feeding jejunostomy was started on the 5th operative day and the patient was discharged on the 16th postoperative day.

**Lessons:**

When a patient under tube feeding exhibits abrupt intractable gastroesophageal reflux with a sign of catheter migration, we must consider the possibility of catheter-related intussusception. Having a fully functioning feeding catheter with nonballoon tip does not preclude retrograde jejunoduodenogastric intussusception.

## 1. Introduction

Retrograde jejunogastric intussusception refers to the invagination of the distal small intestine into the stomach. It is a rare complication following gastrojejunostomy. While such an occurrence is preceded by surgical construction of a direct passage between the jejunum and stomach,^[1,2]^ a true sense of retrograde jejunogastric intussusception, or rather jejunoduodenogastric intussusception, is extremely rare. It is often associated with a displaced gastrostomy tube.^[3]^

Although the exact mechanism remains unclear, retrograde jejunoduodenogastric intussusception is assumed to be preceded by fixation of an inflated tip of a gastrostomy tube onto the mucosa of the small intestine following its migration past the gastric pylorus. Retraction of the migrated catheter (e.g., repositioning attempt) could lead to retrograde invagination of the distal small intestine with the inflated tip acting as a leading point.^[4]^ As such, the tip is often caught within the intussusception or retracted back into the stomach.^[5,6]^ This often results in food intolerance or catheter malfunction among patients with retrograde jejunoduodenogastric intussusception.^[5,7]^

In this report, a distinctive case of retrograde jejunoduodenogastric intussusception associated with a long nonballoon nasojejunal tube while maintaining full catheter function is presented.

## 2. Case presentation

This study was approved by the Institutional Review Board of Kyungpook National University Hospital (Approval No. KNUH 2023-12-009). Written informed consent for publication was obtained from the legal guardian.

A 19-year-old female was presented with repeated vomiting and abdominal distension for 5 days. She was bedridden for 6 months following severe traumatic brain injury. She relied on enteral feeding through a nasojejunal tube, which was inserted by endoscopy and fluoroscopy 2 months earlier. There was no recollection of the tube being pulled hard. However, her caregiver noticed that the tube had been pushed out by several centimeters during routine care several days ago. The exact date was not clear. Not much attention was given at the time because the tube was functioning well. The patient started vomiting (about 1 L per day) 5 days ago. The vomitus was somewhat coffee-ground in color without bile or feeding contents. Having no sign of regurgitation of feeding contents, the enteral feeding continued until the fever occurred a day ago.

The patient had no prior abdominal surgery or allergies. She had a history of major depressive disorder. She had been bedridden since a traumatic brain injury. Her vital signs were as follows: a blood pressure of 158/102 mm Hg, a pulse rate of 178 beats per minute, a respiration rate of 18 breaths per minute, and a temperature of 37.6 °C. Abdominal physical examination revealed severe abdominal distension. Bowel sound was normal. Percussion detected tympanic sounds in the upper abdomen. Pain and tenderness were indeterminable due to underlying conditions related to the previous brain injury. Laboratory studies showed leukocytosis with white blood cell count of 21.0 × 10^9^/L (normal range: 4.8–10.8 × 10^9^/L), elevated C-reactive protein of 7.92 mg/L (normal range: <0.05 mg/L), elevated amylase of 15.00 μkat/L (normal range: 0.47–1.83 μkat/L), and elevated lipase of 27.61 μkat/L (normal range: 0.22–1.00 μkat/L). Levels of total and direct bilirubin were 22.6 μmol/L (normal range: 5.1–17.0 μmol/L) and 14.4 μmol/L (normal range: 1.0–5.1 μmol/L), respectively.

**Figure 1.**
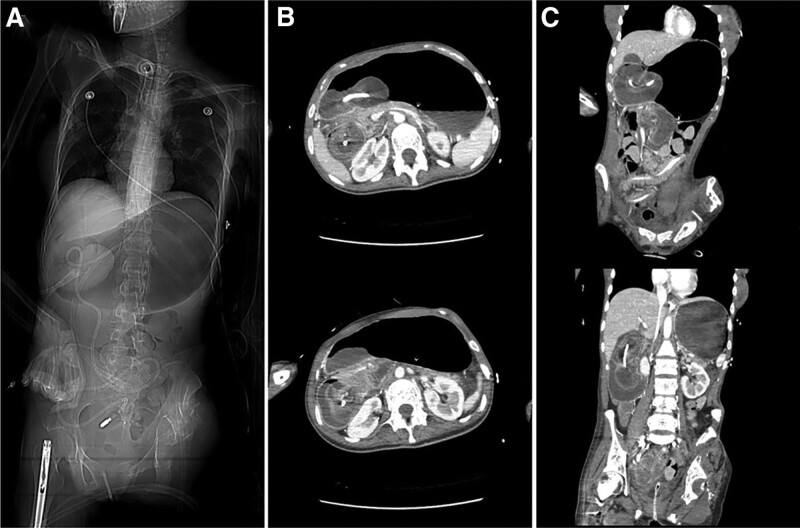
Abdominal computerized tomographic scan taken upon admission. (A) Scout image showing dilated stomach with round opacity in gastric antrum, consistent with migrated small intestine. (B) Axial image and (C) coronal images showing migrated small intestine in gastric antrum with a nasojejunal tube passing through.

**Figure 2.**
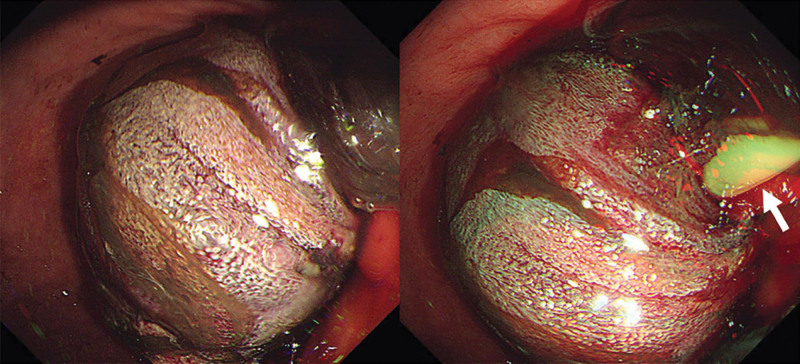
Endoscopic images taken upon admission. A loop of the small intestine was protruding through the pylorus with a nasojejunal tube passing through (single arrow).

The abdominal X-ray image showed a severely distended stomach. An abdominal computerized tomography revealed retrograde jejunoduodenogastric intussusception causing air/fluid-filled gastric distension (Fig. 1). Immediate endoscopic examination was performed. A loop of small intestine protruding through the pylorus was found and progressed ischemia of the migrated small bowel loop was confirmed (Fig. 2). Upon feeling tension during a bedside attempt for catheter removal, no further attempt was made and the patient was sent to the operating room.

**Figure 3.**
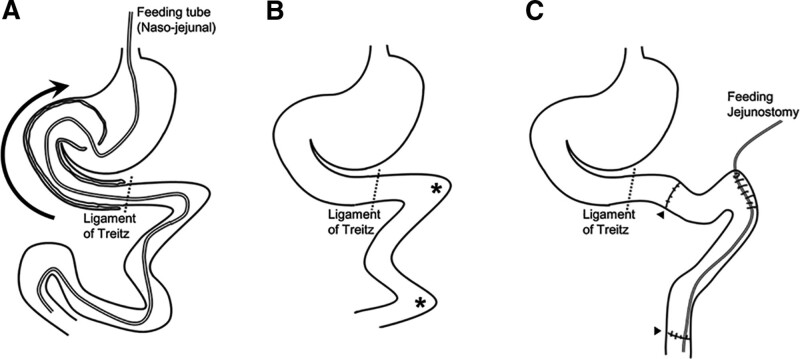
Retrograde jejunoduodenogastric intussusception before/after reduction (A, B) and after surgery (C). Two necrotic segments (asterisk) were resected. After end-to-end anastomoses (arrow head), a feeding jejunostomy was constructed in the proximal jejunum.

At laparotomy, a jejunal loop of 120 cm long migrating into the duodenum and stomach at the level of the ligament of Treitz was noticed (Fig. 3). There was no sign of catheter twist. Upon careful manual reduction, the nasojejunal tube was freed and removed. There were 2 short necrotic segments of the small intestine (5 and 40 cm apart from the ligament of Treitz). Two segmental resections of the necrotic segment followed by end-to-end anastomoses were performed. A feeding jejunostomy was constructed in the proximal jejunum.

Soon after the surgery, levels of serum amylase and lipase started to drop. They became normal on the 2nd operative day. Enteral feeding through the surgically constructed feeding jejunostomy was started on the 5th operative day. The postoperative course was uneventful. The patient was discharged on the 16th postoperative day.

## 3. Discussion

This report presents an adult case of retrograde jejunoduodenogastric intussusception with a fully functioning nonballoon nasojejunal tube. In general, catheter-related intussusception can be divided into antegrade and retrograde intussusception depending on the direction of invagination.^[7]^ The exact mechanisms of each type are unknown. In the case of antegrade intussusception, it has been suggested that a thickened intestinal wall due to repeated irritation from the catheter tip could be the cause. This can result in invagination of the proximal bowel into the distal bowel accompanied by distal migration of the catheter tip.^[8,9]^ Retrograde intussusception has also been suspected to be associated with the preceding distal migration of the catheter and its repositioning attempt.^[7]^ Upon an attempt for repositioning, the distally migrated tip of the catheter can serve as a fishhook and initiate invagination of the distal bowel into the proximal bowel.

The chance of encountering intussusception associated with the stomach in actual clinical practices is extremely rare. There are case reports of antegrade intussusception caused by prolapsed gastric mass into the duodenum.^[10–12]^ There also are reports of retrograde intussusception of the jejunum in the stomach following gastrojejunostomy.^[1,2]^ Occurrence of intussusception involving the stomach without such predisposing factors (i.e., gastric mass or bypass) is even rarer. Except for the above cases, case reports on intussusception associated with the stomach have one thing in common: migrated balloon tip catheter. Few case reports have shown antegrade gastroduodenal intussusception, coinciding with distal migration of the balloon tip feeding catheter.^[13,14]^ Others have shown cases of retrograde intussusception, coinciding with a repositioning attempt of a distally migrated balloon tip catheter.^[3,4,6]^

It has been suggested that loose fixation of the feeding tube onto the abdominal wall could result in migration of the balloon tip.^[15,16]^ Upon anchoring of the bulky balloon onto the gastrointestinal wall, further antegrade or retrograde movement of the tube could result in antegrade or retrograde intussusception. The role of balloon tip as a leading point of the intussusception has been suggested as its de-ballooning has resulted in the releasing of the tethered tube in an invaginated bowel segment.^[17]^ What makes the current case distinctive is that retrograde jejunoduodenogastric intussusception occurred in a patient with a nonballoon tip feeding catheter, defying previous assumptions that the balloon tip might play a crucial role in triggering intussusception. A long-feeding catheter seems to gain enough friction between its lengthy shaft and gastrointestinal mucosa to cause invagination of the intestine upon catheter migration. Moreover, intestinal invagination can occur anywhere along the shaft. Once it occurs at the level of mid-shaft, catheter function might be preserved fully. Unlike previous cases reporting food intolerance or catheter malfunction,^[5,7]^ the patient in the current case remained well-tolerable to tube feeding. Of course, one may raise doubt about the patient being tolerable as her initial presentation was repeated vomiting. However, vomitus was devoid of feeding contents. The catheter was fully functioning without a residual volume and feeding was continued until the day before admission when fever occurred.

Attention needs to be paid to laboratory findings related to obstruction of the ampulla of Vater.^[18,19]^ Regardless of the direction of intussusception (i.e., antegrade or retrograde), a migrated stomach or jejunum may block the pancreatic and bile drainage, resulting in abnormal laboratory findings such as elevated amylase, elevated lipase, or hyperbilirubinemia. While hyperbilirubinemia was not prominent in the current case, serum amylase and lipase showed considerable elevation. Hyperbilirubinemia is expected to occur as the obstruction continues. Resolution of intussusception should lead to normalization of serum laboratory results.

When a patient under tube feeding presents with sudden intractable gastroesophageal reflux, we need to check the positioning of the feeding tube along with the general assessment of the patient tolerance to tube feeding. Evidence of catheter migration or a history of repositioning of the migrated catheter must alert the possibility of catheter-related intussusception. Upon blockage of the biliary tract by a telescoped bowel segment, abnormal laboratory results (e.g., elevated amylase and lipase) may occur. However, a definite diagnosis can be made following an abdominal computerized tomography and endoscopic examination. The endoscopic examination enables direct visualization of the telescoped bowel segment. Surgical exploration should be considered upon detection of irreversible ischemic injury. The strength of the current finding is that it shares a valuable lesson that having a fully functioning feeding catheter without a balloon tip does not preclude retrograde jejunoduodenogastric intussusception. Nevertheless, uncertainty regarding the exact time or event that triggered retrograde intussusception was a limitation of this finding.

## 4. Conclusion

Retrograde jejunoduodenogastric intussusception is extremely rare. It often occurs in association with the migration of feeding catheters. When a patient under tube feeding exhibits abrupt intractable gastroesophageal reflux with a sign of catheter migration, we must consider the possibility of catheter-related intussusception. Having a fully functioning feeding catheter with a nonballoon tip does not preclude retrograde jejunoduodenogastric intussusception.

## Author contributions

**Conceptualization:** Seung Soo Lee.

**Data curation:** Seung Soo Lee.

**Formal analysis:** Seung Soo Lee.

**Investigation:** Seung Soo Lee.

**Visualization:** Seung Soo Lee.

**Writing—original draft:** Seung Soo Lee.

**Writing—review & editing:** Seung Soo Lee.
